# Development of indicators and moral intelligence scales for junior high school students: mixed-method research

**DOI:** 10.1186/s40359-024-01640-w

**Published:** 2024-03-26

**Authors:** Ujsara Prasertsin, Kamontip Srihaset, Pattama Roopsuwankun

**Affiliations:** 1 Faculty of Education, North Bangkok University, 6/999 Soi Phaholyothin 52, Phaholyothin Road, Klong Thanon Subdistrict, Sai Mai District, 10220 Bangkok, Thailand; 2 Educational and Psychological Test Bureau, Srinakharinwirot University, 10110 Wattana, Bangkok, Thailand; 3 Faculty of Education, Ramkhamhaeng University, Ramkhamhaeng Road, Hua-Mark, Bangkapi, 10240 Bangkok, Thailand

**Keywords:** Moral intelligence, Indicators, Norms, Mixed-method research, Junior high school students

## Abstract

The purposes of this research were (1) to study the indicators of moral intelligence for junior high school students, (2) to develop moral intelligence scales for junior high school students, and (3) to assess the needs of moral intelligence for junior high school students. By the mixed-method research, the sample group used in the research was 11 key informants for in-depth interviews, 100 students in mixed classes of 20 schools, 1,997 students in total. The results show the following:

1. The moral intelligence indicators for junior high school students consisted of 6 indicators: Equality, Empathy, Morality, Tolerance, Self-control, and Kindness.

2. To create moral intelligence scales for junior high school students, the Cronbach’s alpha coefficients for each section were.939,0.920 and.841, respectively. With corroborative component analysis classified by the overall composition, it was found that the mean was at a high level (M = 3.955), with a low distribution with a standard deviation (SD) of 0.576 and a distribution coefficient (C.V.) of 14.566.

3. For needs assessment of moral intelligence for junior high school students, it was found that the third component of morality had the most needs (PNI_modified_=0.095), followed by the 5th component of self-control (PNI_modified_=0.081), the second component of empathy (PNI_modified_=0.075), the 4th component of tolerance (PNI_modified_=0.073), the 6th component of kindness (PNI_modified_=0.055) and the first component of equality (PNI_modified_=0.053).

**Supplementary Information:**

The online version contains supplementary material available at 10.1186/s40359-024-01640-w.

## Introduction

Enhancing ethical intelligence is an important factor for human beings to learn from experience to think critically about solving problems in different situations. This can be applied in a normal life because ethical intelligence is one of the factors that help encourage learners. It is a part of holistic education in helping develop a person’s abilities, consciousness, conscience, discrimination, good and bad, and social existence. It also builds relationships and ethical intelligence behaviors in educational institutions and develops the potential of a person after graduation to pursue a career (Rodney, [1]; Malikeh, et al., [2]). “Moral Quotient” (MQ) or “Intelligence in Ethics” or “Ethical Intelligence,” a Thai word, (Suphanphet, [3]) and “intelligence in doing good deeds or moral intelligence” (Kusiriwichian, [4]) all refer to the same meaning. Both can be interpreted as ethical intelligence depending on the context of the research and the researcher. However, most foreign researchers use the term “moral intelligence,” MI. According to the study of principles, concepts and theories of ethical intelligence, it refers to the level of moral ethics of a person who can understand self-control as an intellectual center by setting rules to think and act in the right way with honesty, compassion, forgiveness, and self-responsibility toward society and humanity (Lennick & Kiel, [5]; Nixon, [6]). As a result of one’s own actions, everything having been done follows the law of cause and effect (the law of cause and effect), consistent with the research of (Teo & Lachlan, [7]). For a person’s good level of ethical intelligence, it must be done in early childhood nurture because this will enable a person to develop and instill love. This is an important key to developing ethical foundations for making decisions to behave according to righteousness with good intentions in every situation (Borba, [8]; Lennick & Kiel, [9]). According to former studies, ethical intelligence has only been associated with abstract and concrete individuals whose rationale and ethical behaviors remain unclear. Currently, research on ethical intelligence, especially overseas studies, has been studied and researched in this aspect by expanding the descriptions and relevant components based on the development of moral intelligence structure (Development of Moral Intelligence Structure) (Sokhuma, [10]).

Therefore, to effectively develop ethical intelligence scales for junior high school students, the researcher has added several methods for gaining knowledge from research on needs. One important method is ‘needs assessment’ and ‘needs assessment research,’ a systematic process to specify differences between expected conditions and actual conditions. The data have been used to prioritize the differences and then select the critical needs to determine causes and assessment guidelines. In needs assessment, the results of needs assessment must be prioritized at every step of the process (Wongwanich, [11]).

For the reasons mentioned above, the purposes of this research were to develop moral intelligence scales for junior high school students by using mixed-method research, relating to a link between quantitative and qualitative research to access data comprehensively. The results of this research will be beneficial for the self-improvement process leading to social development, especially for junior high school students, who will grow up as an important workforce to develop the nation in the future.

## Methodology

The researcher designed mixed-method research between quantitative and qualitative methods consisting of three phases of methodology based on three objectives. In Phase 1 and Phase 2, the researcher designed the research in the form of an Exploratory Design: instrument development model (Creswell & Plano Clark, 2007; Prasertsin, [12]) as the creation of quantitative tools with qualitative findings, the use of qualitative data to create and design tools used quantitatively in Phase 2. In Phase 3, the researcher designed the research to assess needs by designing each phase as a Triangulation Design: Convergence Model. The steps of each phase were detailed as follows:

### Phase 1 Study of ethical intelligence indicators for junior high school students

In the study of the ethical intelligence indicators for junior high school students, relevant literature and research were reviewed to define and identify indicators of ethical intelligence for junior high school students among domestic and overseas studies to obtain data as accurate as possible in today’s Thai society. Specifying qualitative ethical intelligence indicators for junior high school students was studied through in-depth interviews with a total of 1 key informant from schools in various regions of Thailand. An interview questionnaire was used as a tool to gain data on the students’ ethical intelligence. The researcher has studied related documents and studies to create a conceptual framework and select case studies.

### Phase 2 Development of ethical intelligence scales for junior high school students

To create ethical intelligence scales for junior high school students, the researcher used the ethical intelligence indicators for junior high school students obtained from Phase I to create and design a tool for quantitative use. The survey was conducted with 1,997 junior high school students from various regional schools nationwide. To examine the coherence of the ethical intelligence measurement model for junior high school students, the researcher conducted a corroborative component analysis (CFA) of the data collected from Phase 2 to test the validity of the data obtained from the phase 1 indicators.

### Phase 3 Needs assessment of ethical intelligence for junior high school students

Data of the measurement from Phase 2 were measured with 1,997 junior high school students from various regional schools nationwide to analyze and assess the needs of ethical intelligence for junior high school students.

#### Tools and quality testing

The researcher verified the validity and reliability of the data by triangulation (Denzin, 1970 cited in Chantavanich, [13]) to examine the sources of the data.

#### Method of data collection

The researcher planned to use the data obtained from the collection process to reduce, check and analyze the data. These 3 processes were conducted in parallel with the data collection process, and the last step was to analyze the data for qualitative study.

#### Quantitative data analysis

The researcher used (1) primary data analysis to describe general characteristics of categorical data such as frequency distribution, numbers, percentage, and descriptive statistical analysis to present primary statistical values of continuous variables (continuous data) to see distribution characteristics and a distribution of variables, (2) data analysis to examine indicators and quality of ethical intelligence scales for junior high school students with a corroborative component analysis (CFA), and (3) needs analysis with index values PNI_modified_=(I-D)/D to manipulate needs in descending priorities through the PNI_modified_ index. A high index referred to high needs for more development than a lower index (Wongwanich, [11]).

#### Qualitative data analysis

The researcher used (1) content analysis from participant observation, informal interviews, in-depth interviews, and documentary analysis and (2) data classification to classify into categories or types based on the Lofland Concept of Social Phenomena Observation (1971 cited in Chantavanich, [13]) as classification criteria.

## Results

### Study of ethical intelligence indicators for junior high school students

It was found that ethical intelligence indicators referred to a person’s intellectual ability to learn, behave and make decisions about what to do or not do in society. A level of individual moral intelligence consisted of 6 indicators: (1) Equality referred to respecting others’ differences, not discriminating, equality and not taking advantage; (2) Empathy referred to empathy for others, emotional empathy, responsibility for duties; (3) Morality referred to adherence to righteousness, observance of social doctrines and rules, as peaceful as coexistence with basic morality; (4) Tolerance referred to insensitivity to provocative stimuli, perseverance, and determination; (5) Self-control referred to emotional self-regulation with firmness, self-mental control of unwanted emotions and behaviors; and (6) Benevolence referred to good will to help others according to one’s own strength, no oppression and common good thinking.

The researcher created and designed quantitative tools for lower secondary students using ethical intelligence indicators obtained from Phase 1.

### Development of ethical intelligence scales for junior high school students

The researcher created and designed quantitative tools for junior high school students using the ethical intelligence indicators obtained from Phase 1. A survey was conducted with 2,000 junior high school students from various regional schools nationwide. Tool quality testing was conducted for the content validity of the indicators used to measure the ethical intelligence of the students. Definitions of each component were checked and recommended by 5 experts in the conformity of the components and definitions by calculating the Index of Item Objective Congruence (IOC) for each question. The Index of Item Objective Congruence (IOC) must be between 0.60 and 1.00. The questionnaire was then used to test the content validity of the tools (Try Out) with 55 junior high school students in the population group, but not subjects, to determine the quality of reliable tools (Reliability). To measure variables used in the research by using Cronbach’s alpha coefficient formula (Prasertsin, [14]), the results of the study in this section showed values for the reliability of each section and each component as follows: (1) Part 2, Questions of Opinions and Practices had 6 items on each aspect, 36 items in total, with a validity value of the whole text α = 0.939; (2) Part 3, Reasoning Situational Questions included 6 conditions, 24 items, with validity values α = 0.920; and (3) Part 4, Behavioral Situation Questions included 6 situations, 24 items in total, with a reliability value of the whole text α = 0.841.

To examine the coherence of the ethical intelligence measurement model for junior high school students, the results were as follows: Part 1, basic data of junior high school students, showed that the majority of students were 60.94% female. Most of them were studying in Mathayom 2, accounting for 41.71%. In consideration of overall GPA, it was found that junior high school students had a GPA of 3.299, with a small distribution, a standard deviation (SD) of 0.571 and a dispersion coefficient (C.V.) of 17.316. When considering the distribution of data, the negative skewness (Sk) and the negative kurtosis (Ku) indicated that the majority of the samples had overall above-average GPAs and a high distribution of data. The results of the reviewed ethical intelligence model for junior high school students revealed that the construct validity of ethical intelligence scales for junior high school students was examined by analyzing the construct validity of the ethical intelligence model for junior high school students through confirmatory factor analysis (confirmatory factor analysis). The details were as follows: The results of the data analysis on moral intelligence variables for junior high school students classified by components as a whole showed that the mean was at a high level (M = 3.955), with a small distribution, a standard deviation (SD) of 0.576 and a dispersion coefficient (C.V.) of 14.566. When considering the distribution of the data, the negative skew (Sk) and the negative Ku (Ku) indicated that the majority of the samples had above-average moral intelligence scores and a high distribution of the data. In consideration of each component, it was found that all components had a high mean, with Component 1 Equality having the highest mean, followed by Component 2 Empathy, Component 6 Kindness, Component 5 Self-Control, Component 4 Tolerance and Component 3 Morality (M = 4.088, 3.999, 3.988, 3.931, 3.895 and 3.832, respectively). All components had a slight distribution with a standard deviation (SD) between 0.610 and 0.685 and a distribution coefficient (C.V.) between 14.917 and 17. 219. The most distributed component was Component 4, Tolerance. The least distributed component was Component 1 Equality. When considering the distribution of the data, the negative skew (Sk) and the negative Ku (Ku) indicated that the majority of the samples had higher than average ethical intelligence scores in each component and a high distribution of the data.

The correlation coefficient matrix analysis of 15 pairs of observed variables in a model was different from zero at a statistical significance level of.05 (p =.000). In all pairs, the correlation coefficient was between 0.642 and 0.776. In terms of correlation size, it was found that the correlation variables were at a moderate level. The observed variable with the highest mean was Component 1, equality (M = 4.088, SD = 0.610), and the observed variable with the lowest mean was Component 3, morality (M = 3.832, SD = 0.651). When considering the results of Bartlett’s test of sphericity, a statistical test hypothesis whether the correlation matrix was an identity matrix, χ^2^ = 10019.858 (df = 15, p =.000), was significantly different from zero at a statistical significance level of.01 and consistent with the index analysis results of Kaiser‒Meyer‒Olkin (KMO), approaching 1 (KMO = 0.925). It revealed that the observed variable correlation matrix was not an identity matrix and that there was enough correlation between the variables for component analysis to verify structure validity. The details of the means, standard deviations and correlations of the observed variables were obtained in the ethical intelligence measurement model for junior high school students.

The results of the corroborative component analysis revealed that the ethical intelligence model for junior high school students was consistent with the empirical data, as determined by GFI = 0.918, AGFI = 0.906, NFI = 0.924, RFI =. 0.917, IFI = 0.939, TLI = 0.934, RMR = 0.025, RMSEA = 0.042 and CFI = 0.939. The coherence indexes were in accordance with the criteria of Hair et al. [15]. The standard component weight coefficient (β) of the observed variables or indicators showed that all components of the indicators had a standard component weight coefficient (β) of the observed variables or indicators. All of them were statistically significant at the.05 level (p =.000).

The results of the preliminary statistical analysis of moral intelligence scores for junior high school students with reasoning situational questions classified by components presented showed that the overall analysis results were at a high level (M = 3.958), with a low distribution, a standard deviation (SD) of 0.712 and a distribution coefficient (C.V.) of 17.985. When considering the data distribution, it was found that the negative skew (Sk) and the negative Ku (Ku) indicated that the majority of the samples had ethical intelligence scores for junior high school students. For reasoning situation questions, it was above average, and the data had a high distribution. Considering each component, it was found that all components had high means. For the behavioral situation questionnaire, it was found that the mean was 58.260 (M = 58.260), with a fairly large distribution, a standard deviation (SD) of 6.778 and a distribution coefficient (C.V.) of 11.635. When considering the distribution of the data, it was found that the negative skew (Sk) and the positive Ku (Ku) indicated that the majority of the samples had ethical intelligence scores for junior high school students. The overall behavioral situation questions were above average, and the data had a slight distribution. For the results of the correlation coefficient matrix analysis, all 3 ethical intelligence scores for junior high school students were among scores from opinion and action questions, scores for situational rationale questions, and scores from questionnaires. Three pairs were different from zero at a statistical significance level of 0.05 (p =.000). In all pairs, the correlation coefficient was between 0.257 and 0.678. In terms of correlation size, the correlation of variables was at a low to moderate level.

### Ethical intelligence needs assessment for junior high school students

Current and expected needs were prioritized using the PNI_modified_ Priority Needs Index method, calculated from a mean of the expected condition (I) and a mean of the actual condition (D). No. 1 compared to each component revealed that Component 3 Morality had the highest need (PNI_modified_=0.095). No. 2 compared to 36 items in total revealed that item 17, the students told the truth reasonably under good morals, had the highest need (PNI_modified_=0.122). Considering lists of items of Component 1 Equality, it was found that item 5, communicating with others with a sense of conscience, was what students were most aware of in their lives (PNI_modified_=0.082). For component 2, Empathy, item 7, students with careful thinking before communicating or talking to others to not make others lose their feelings, had the highest need (PNI_modified_=0.104). Component 3 Morality found that in item 17, students spoke truthfully and rationally under good morals (PNI_modified_=0.122). Component 4, Tolerance, found that item 24, when encountering various events in their lives, students were able to be patient and wait, had the highest need. (PNI_modified_=0.093). Component 5, self-control, revealed that item 28, when a mistake occurred, students did not blame others or blame situations, was the most imperative (PNI_modified_=0.101). Component 6, Kindness, revealed that item 36, students focused on doing good for the public to make people around happy, had the highest need (PNI_modified_=0.083).

## Discussion

### Study of ethical intelligence indicators for junior high school students

It was found that ethical intelligence indicators referred to characteristics of a person’s intellectual ability to learn, behave and make informed decisions on what to do or not to do in society. It was a level of a person’s moral intelligence consisting of six indicators: (1) equality, (2) empathy, (3) morality, (4) tolerance, (5) self-control, and (6) kindness. This was consistent with the model of Borba [8], an educational psychologist who gave the idea of ethical intelligence and presented seven components of ethical intelligence enhancement: (1) empathy, (2) conscience, (3) self-control, (4) respect (5) kindness, (6) tolerance, and (7) fairness. In addition, Lennick & Kiel [9] divided the core components of ethical intelligence into four aspects: honesty and integrity, responsibility, compassion, and forgiveness, which were used to assess a person’s level of ethical intelligence. Consistent with Clarken’s [16] study of ethical intelligence in schools, it was found that ethical intelligence referred to the ability to apply ethical principles to personal goals, values, and actions. The structure of ethical intelligence consisted of four competencies related to honesty, responsibility, forgiveness, and compassion. This finding was consistent with research by Prasetiawan & Barida [17], who studied improving adolescents’ moral intelligence and practical problem-solving approaches by using an ethical intelligence tool that consisted of 7 aspects: empathy, self-control, conscience, respect, kindness, tolerance and fairness.

Although the indicators in each theory and research have different components, they all have a comprehensive meaning.

### Development of ethical intelligence scales for junior high school students

To conduct a tool quality assessment for consistency of the indicators used to measure ethical intelligence of students, the researcher provided (the) definitions of each component for 5 experts to verify their conformity and definitions by calculating the Index of Item (IOC) of each item (The Index of Item Objective Congruence; IOC) between 0.60 and 1.00. It was shown that an ethical intelligence model for junior high school students was created as ethical intelligence behavior alternatives with high content validity, in accordance with the principles of Srisa-ard [18]. It stated that a consistent index of 0.50 or higher was considered a good, measurable, comprehensive and representative behavioral trait. Therefore, it can be said that the ethical intelligence model for junior high school students created by the researcher was consistent with the content validity of tool quality on reliability (reliability).

To measure the variables, the researcher used Cronbach’s alpha coefficient formula corresponding to the criteria for determining the acceptable level of reliability. It should be at 0.70 or higher (Pinyoanuntapong, [19]). This is consistent with Johnson and Christenson, who recommend that scales should have a reliability coefficient of 0.70 or higher, depending on the research objective (Mitranun et al., [20]). The results of the corroborative component analysis revealed that the ethical intelligence model for junior high school students was consistent with the empirical data as determined by GFI = 0.918, AGFI = 0.906, NFI = 0.924, RFI =. 0.917, IFI = 0.939, TLI = 0.934, RMR = 0.025, RMSEA = 0.042 and CFI = 0.939. The harmonization index satisfied the criteria of Hair et al. [15]. When the results of the analysis are compared with the model-to-empirical coherence criteria stated by Joreskog and Sorbom [21], it was said that the model was consistent with the empirical data when the RMSEA and standard RMR values were less than 0.05. The GFI and AGFI values should be greater than 0.90. It was found that the data were in accordance with specified criteria, whereas the Harmony Level Index was in accordance with the criteria of Hair et al. [15]. For all pairs of variables, the correlation coefficient was between 0.257 and 0.678. The correlation of the variables was low to moderate, meaning that a data set was suitable for elemental analysis, as it had no more than 0.8 parts.

### Needs assessment of ethical intelligence for junior high school students

It was found that Component 3 Morality was the highest need (PNI_modified_=0.095), consistent with key principles of educational psychologists such as Freud [22], Piaget [23] and Skinner [24]. They emphasized morality as a social cognitive phenomenon. Individuals developed concepts of righteousness or conscience in the context of social interaction and awareness of consequences reflected through the actions and reactions of people in society. It was found from the study that items on the topic of telling the truth were subject to good morals. Conscious communication with others was a realization among students in their lives. Thinking carefully before communicating or talking with others in order not to let others feel bad. In addition, when things came into their lives, students could be patient and wait, including when mistakes happened. Students were not about to blame others or situations, and they focused on doing well for the public to make people around them happy. These were parts of emotional intelligence. That could help people understand themselves and others’ emotions and control their thoughts and actions. It was a person’s strength in learning new knowledge, problem solving, interpretation, and abstract thinking, as well as creating more creativity. Creativity could also lead to efficiency in thinking. Empowering individuals with emotional intelligence was a preliminary step for later developing and improving creativity. Happiness and emotional intelligence could bring positive emotions to a person. and the result is more positive performance (Mehmet, et al., [25]).

These topics were the highest need, consistent with Lind [26], who noted that ethical competence, including accountability, referred to the process by which a person owned his or her own actions and accepted the fact that he or she was responsible for all consequences arising from such actions, including taking responsibility for personal choices, acknowledging one’s own mistakes and failures, and accepting responsibility for serving others to show compassion for others. All of these actions reflected respect for fellow human beings. Engaging in others’ daily lives was an expression of concern that fostered mutual trust and partnership, consistent with Lennick & Kiel [5]. They stated that honesty, responsibility, compassion and forgiveness were universal human principles that could not be changed in any gender, race, culture or religion. It was emphasized that personal behaviors were demanded to change in accordance with these universal principles. This is also consistent with Monir’s [27] research, which found that there was a statistically significant relationship between self-compassion and kindness (self-compassion) and moral intelligence. There were some aspects of moral intelligence, such as honesty, responsibility, sympathy and forgiveness. These dimensions were closely related to self-compassion. Therefore, when self-compassion was developed, it would develop moral intelligence. Educators could integrate self-compassion into their teaching curriculum and connect it with moral intelligence.

### Recommendations

1. The components and indicators of ethical intelligence should be studied for youth in other groups, such as primary school students, senior high school students, university students, vocational students, etc., including the development of other indicators as complementary factors (family support/social contributors) for the ethical intelligence scale model and indicators from this study.
2. The data obtained from the critiques by the expert group should be studied in order to gain more results of the research and develop the ethical intelligence measurement for students in other grades or do further in-depth studies and then make improvement of the measurement model more standardized.
3. The research findings should be studied to broaden the perspectives of parents, educators and community leaders on the components and indicators of ethical intelligence to use the results to further develop ethical intelligence scales.

### Electronic supplementary material

Below is the link to the electronic supplementary material.

Supplementary Material 1

## Data Availability

The datasets generated and/or analyzed during the current study are not publicly available because permission was not obtained from the participants to share their data publicly but could be available from the corresponding author on reasonable request.
