# Supplementary material for: Development of indicators and moral intelligence scales for junior high school students: mixed-method research
Source: BMC Psychol. 2024 Mar 26;12:174. doi: 10.1186/s40359-024-01640-w (PMC10964494; doi:10.1186/s40359-024-01640-w)
Supplement: Supplementary file 1 — Supplementary Material 1 [file 40359_2024_1640_MOESM1_ESM.docx]

**Interview form**

**Phase 1: Study of indicators of moral intelligence for Junior High School Students.**

**Subject: Development of Indicators and Moral Intelligence Scales**

**for Junior High School Students: Mixed – Method Research**

Interviewee……………………………Position………………………...

Workplace……………………….…Work experience ...................year

1. Moral intelligence of students at the junior high school student level in your opinion, what does it mean? and how important is it in today's era?

………………………………………………………………………………………………………………………………………… ………………

2. Moral intelligence of students at the junior high school student level that the research team has implemented consists of 6 elements: (1) Equality, (2) Empathy, (3) Morality, (4) Tolerance, (5) Self-control and (6) Kindness and compassion Do you think it is appropriate or not? What changes should be made?

………………………………………………………………………………………………………………………………………… ………………

3. From item 2, there are 7 components of moral intelligence. What meaning should each component have?

(1) Equality

………………………………………………………………………………………………………………………………………… ………………

(2) Empathy

………………………………………………………………………………………………………………………………………… ………………

(3) Morality

………………………………………………………………………………………………………………………………………… ………………

(4) Tolerance

………………………………………………………………………………………………………………………………………… ………………

(5) Self-control

………………………………………………………………………………………………………………………………………… ………………

(6) Kindness

………………………………………………………………………………………………………………………………………… ………………

4. What guidelines do you think there should be for creating or developing criteria and a manual for measuring the moral intelligence of junior high school students? Please specify the nature of the criteria and manual including necessary information.

………………………………………………………………………………………………………………………………………… ………………

5. What do you think should be a tool for measuring moral intelligence that is appropriate for the development according to the age of junior high school students?

………………………………………………………………………………………………………………………………………… ………………

6. If the results from measuring the moral intelligence of junior high school students are to be used to develop and promote both study skills and living in society, including the lives of students. How can it be used? What other techniques should be used to help promote it?

………………………………………………………………………………………………………………………………………… ………………

7. What factors (both supporting factors and hindering factors) play an important role in the application of the results of the moral intelligence assessment of lower secondary school students? Who should play a role in managing that factor? And what are the guidelines for taking action?

………………………………………………………………………………………………………………………………………… ………………

**** Thank you everyone for giving up valuable time for the interview ****
